# The Growth of the Nursing Profession. II. Become Founders and Helpers of Democratic Government

**Published:** 1918-01-26

**Authors:** 


### THE GROWTH OF THE NURSING PROFESSION.

### II.—Become Founders and Helpers of Democratic Government.

The immediate events which led to the establishment of the College of Nursing are now so well known that it is unnecessary to reiterate them. The College is an established fact, acknowledged and welcomed by the nurses, by the teachers of nurses, and by the public. That furious controversy accompanied by bitter calumny and fierce invective should rage around it in its early years is but natural, and is rather a sign of health and vitality than of any intrinsic defect in the idea itself. What is the underlying idea of the College of Nursing? It is, that there should be, instead of associations and societies innumerable, a single representative body speaking for the nurses and in the name of the nurses. Doubtless the idea is not an entirely original one. Many an inventor has received the credit for an invention which was already existent in some other man's brain, simply because he has acted upon his knowledge of and his belief in the value of his idea. The founders of the College of Nursing have thus acted upon their belief in the possibilities of the idea of a single representative body for the nursing profession, and to-day there are in consequence some seven thousand nurses openly proclaiming that they share this belief.

Given this central idea as a basis, let us, avoiding all side issues—of which there are countless hosts—see how it ought to work out. The primal necessity is that all nurses should be brought together, and the many voices now speaking be harmonised in one authoritative utterance. For this reason the first step to be taken by any body working on this basis is naturally to make a list, roll, register—by whatever name we choose to call it—of nurses who by virtue of having attained certain standard qualifications have earned the right to have a voice in the affairs of their profession. Hence we get the Register of the College of Nursing, which in the midst of the whirlwinds that have stormed around the various side issues, has still been its first care. Quietly and steadily it has increased in length, and were it not for those same side issues the College Register would to-day number practically the whole profession. As it is, it has a membership at least twice as large as any other nursing association, and is therefore sufficiently far advanced towards the realisation of its central idea—which must always be kept clearly before us—to enable it to take the next step. This next step is, that all these nurses being thus brought together should choose their own leaders. This in actual fact will be taking place in the coming month of March: a proportion of the present College Council, which has done the work of enrolling the nurses, will retire from office, and their successors will be chosen and elected by the nurses now on the College Register. These members will thus be able to claim that they speak on behalf of some seven thousand nurses, and are therefore undoubtedly entitled, not only to a hearing, but to be entrusted with the work for which a representative body was needed.

We do not wish to enter into the discussion of any side issues whatever, but one fact might be pointed out which will help us to keep our central idea still clearly before us. Seven thousand is not a majority of the nurses of Great Britain and Ireland. What of the other thousands? Not more than four—obviously a minority—are articulate. The remaining thousands have not chosen to join the self-governing nurses' associations. If they remain aloof and do not promptly join the College of Nursing, they deprive themselves of the right to speak for their profession or to expect any advance on its part. It must be assumed that they take no real interest in their profession as such, but confine themselves entirely to the daily task which is before them, oblivious of larger issues and careless of the generations to follow. Therefore, if they do not choose to speak, they cannot expect to be taken into account. From this it follows that the organisation which has the largest membership has the right to speak and work as representing the profession, and if its words and works do not meet with the approval of these inarticulate members, they have no justification for resentment: had they joined it they could have helped to mould its works in the form which they thought best. Trained nurses are joining the College in increasing numbers every day, clear proof that members of the profession have become alive to the privilege of taking part in the first election for members of its Council. All who join now will be founders and helpers from the outset of democratic government.

It is therefore with confidence in its representative claims that we can proceed to investigate the work that lies before the College, and the Council which its members will have elected.

☛ The previous article appeared on January 19, 1918.

